# The natural history of QTc interval and its clinical impact in coronavirus disease 2019 survivors after 1 year

**DOI:** 10.3389/fcvm.2023.1140276

**Published:** 2023-04-06

**Authors:** Diana Mojón-Álvarez, Andrea Izquierdo, Héctor Cubero-Gallego, Alicia Calvo-Fernández, Jaume Marrugat, Silvia Pérez-Fernández, Paula Cabero, Claudia Solà-Richarte, Cristina Soler, Núria Farré, Beatriz Vaquerizo

**Affiliations:** ^1^ Cardiology Department, Hospital del Mar, Barcelona, Spain; ^2^ Medicine Department, Autonomous University of Barcelona, Barcelona, Spain; ^3^ IMIM, Heart Disease Biomedical Research Group, Barcelona, Spain; ^4^ Medicine Department, Pompeu Fabra University, Barcelona, Spain; ^5^ CIBER Group in Epidemiology and Public Heath (CIBERCV), Hospital del Mar Medical Research Institute (IMIM), Barcelona, Spain; ^6^ REGICOR (Registre Gironí del Cor) Study Group, IMIM (Hospital del Mar Medical Research Institute), Barcelona, Spain; ^7^ Scientific Coordination Facility, Biocruces Bizkaia Health Research Institute, Barakaldo, Spain

**Keywords:** COVID-19, electrocardiogram (ECG), arrhythmia, QTc interval, mortality

## Abstract

**Background and objective:**

Prolonged QTc interval on admission and a higher risk of death in SARS-CoV-2 patients have been reported. The long-term clinical impact of prolonged QTc interval is unknown. This study examined the relationship in COVID-19 survivors of a prolonged QTc on admission with long-term adverse events, changes in QTc duration and its impact on 1-year prognosis, and factors associated with a prolonged QTc at follow-up.

**Methods:**

We conducted a single-center prospective cohort study of 523 SARS-CoV-2-positive patients who were alive on discharge. An electrocardiogram was taken on these patients within the first 48 h after diagnosis and before the administration of any medication with a known effect on QT interval and repeated in 421 patients 7 months after discharge. Mortality, hospital readmission, and new arrhythmia rates 1 year after discharge were reviewed.

**Results:**

Thirty-one (6.3%) survivors had a baseline prolonged QTc. They were older, had more cardiovascular risk factors, cardiac disease, and comorbidities, and higher levels of terminal pro-brain natriuretic peptide. There was no relationship between prolonged QTc on admission and the 1-year endpoint (9.8% vs. 5.5%, *p* = 0.212). In 84% of survivors with prolonged baseline QTc, it normalized at 7.9 ± 2.2 months. Of the survivors, 2.4% had prolonged QTc at follow-up, and this was independently associated with obesity, ischemic cardiomyopathy, chronic obstructive pulmonary disease, and cancer. Prolonged baseline QTc was not independently associated with the composite adverse event at 1 year.

**Conclusions:**

Prolonged QTc in the acute phase normalized in most COVID-19 survivors and had no clinical long-term impact. Prolonged QTc at follow-up was related to the presence of obesity and previously acquired chronic diseases and was not related to 1-year prognosis.

## Introduction

The world has been experiencing a pandemic since December 2019 from a novel coronavirus, responsible for the Severe Acute Respiratory Syndrome Coronavirus 2 (SARS-CoV-2), which has caused great morbidity and mortality throughout the world (1). Although the effects of coronavirus disease 2019 (COVID-19) are predominantly respiratory, some cardiovascular effects, like nonspecific myocardial damage, heart failure, QTc prolongation, and arrhythmias have been described (2–4). Some studies have reported an association between QTc prolongation on admission and higher mortality risk in hospitalized COVID-19 patients (5, 6). A significant relationship between systemic inflammation, QTc prolongation, and arrhythmias during the acute phase of infections has been demonstrated in both COVID and non-COVID-19 patients (7, 8). The characteristic systemic inflammation caused by COVID-19 disease (“cytokine storm”) is likely a crucial factor in QTc prolongation by several mechanisms (9–11).

There is little information on the long-term consequences of COVID-19 in patients after hospital discharge. Most studies have been limited to hospitalized individuals, and all had a short duration of follow-up and narrow selection of cardiovascular outcomes (12–14). Recently, a large study showed that, beyond the first 30 days of infection, people with COVID-19 exhibited increased risk for and 12-month burden of cardiovascular diseases (15).

Considering the prognostic role of a prolonged QTc at hospital admission and availability of electrocardiogram (ECG) in any healthcare setting, ECG could be a potential tool to identify high-risk patients. Thus, we aimed to describe the long-term impact of a prolonged QTc on admission in COVID-19 survivors and identify potential risk factors related to a prolonged QTc at follow-up.

## Materials and methods

### Patient population and data collection

**Figure 1:**
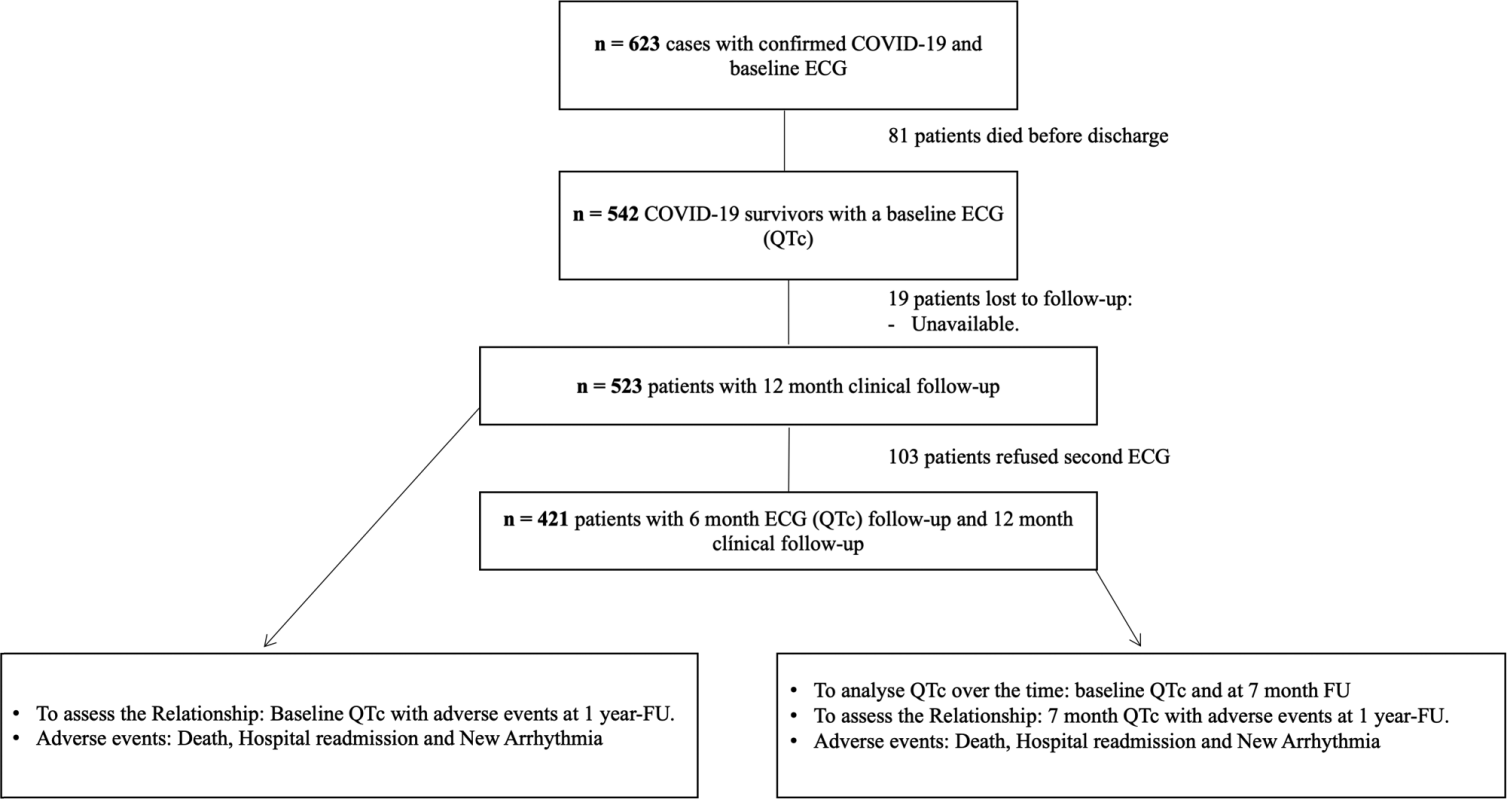
Flowchart of patient inclusion.

A single-center (Hospital del Mar—Barcelona, Spain) prospective cohort study of consecutive patients hospitalized with COVID was conducted. The acute impact of prolonged QTc interval on admission in SARS-CoV-2 patients has been published, and trial design and results have previously been described in detail (5). In brief, our initial cohort included a total of 623 consecutive patients with polymerase chain reaction test (PCR) positive to SARS-CoV-2 recruited from 27 February to 7 April 2020. An ECG was taken on these patients within the ﬁrst 48 h after diagnosis and before the administration of any medication with a known effect on QT interval. Baseline ECG was deﬁned as the ECG taken within the ﬁrst 48 h after laboratory-conﬁrmed COVID-19 diagnosis and before the administration of any medication with a known effect on the QT interval. At the time of the pandemic, our institutional protocol recommended an ECG before starting any medication with a known effect on QT interval. Prolonged QTc was deﬁned as a QTc≥480 ms (16, 17). From this initial cohort, patients who were alive upon discharge were included in this second study. Five hundred and forty-two patients with a baseline ECG were discharged alive during the study period. Nineteen patients were lost to follow-up. Therefore, the final sample was 523 patients with clinical follow-up. Of those, 80.5% consented to a repeat ECG at 7-month follow-up (Figure 1).

Demographic characteristics (age and sex), comorbidities, laboratory parameters, chest radiography, electrocardiographic ﬁndings, treatments, complications, and outcomes were collected prospectively using an electronic data capture system. Patients had an in-person follow-up for repeat ECG and blood test analysis at 7 months. One-year follow-up was performed by telephone contact and through electronic medical records.

This study was performed according to the principles of the Declaration of Helsinki, ISO14155, and clinical practice guidelines. The study protocol was approved by the Institutional Ethics Committee and the hospital research committee. Informed consent was obtained from all patients.

### Electrocardiogram and QTc interval

Standard resting 12-lead ECG was obtained in all patients using the Philips PageWriter TC30 Cardiograph (Koninklijke Philips, Eindhoven, Netherlands). Two ECGs were obtained. The first one was performed within the ﬁrst 48 h after laboratory-conﬁrmed COVID-19 diagnosis. We considered 6 months as the time enough to normalize the QTc interval if its prolongation was related with SARS-CoV-2 infection. The second ECG was performed on the COVID-19 survivors who agreed to come to the hospital after discharge. For organizational reasons, we started our follow-up at 5.7 months, with a mean follow-up of 7.9 months.

The following ECG parameters were calculated: QRS interval (ms), QT interval, and QTc that were automatically calculated as the time from the start of the Q wave to the end of the T wave and corrected for heart rate by the Bazett formula (QTc) in ECG I and ECG II. All the measures were manually revised and checked by trained researchers. In the setting of a broad QRS complex, we used the Bogossian formula to correct the QT. Prolonged QTc was deﬁned as a QTc ≥480 ms (17). A baseline QTc <480 ms and QTc≥480 at 7 months was named new prolonged QTc at follow-up. A baseline QTc≥480 ms that continued to be prolonged (QTc ≥480 ms) at 7 months was named persistent prolonged QTc at follow-up. A baseline QTc ≥480 ms that progressed to normal (QTc < 480 ms) at 7 months was named normalized QTc at follow-up.

### Endpoints and definitions

The primary endpoint was a composite of death, new episode of arrhythmia, or all-cause hospital readmission at 1 year from initial hospital admission for COVID-19. The secondary endpoints were to describe the temporal evolution of the QTc interval and its relationship with prognosis and factors associated with QTc changes over time.

A new arrhythmia episode was defined as any new ECG-confirmed diagnosis of supraventricular or ventricular arrhythmia that was not present on the baseline ECG. Obesity was defined as a body mass index (BMI) over 30 kg/m^2^.

### Statistical analysis

Continuous variables were expressed as the mean and SD except when not following the normal distribution, in which case they were expressed as the median and 25–75th percentile. Normal distribution was assessed using the Shapiro–Wilk test and normal Q–Q plot. Categorical variables were expressed as number and percentage. Differences between groups were assessed using Student's *t*-test or Mann–Whitney *U* test, as appropriate, for continuous variables and the Chi-square test for categorical variables. Differences between baseline and 7-month QTc were assessed using paired Student's *t*-test for continuous variables and the McNemar test for categorical variables. Kaplan–Meier survival curves for the composite endpoints were plotted, and the log-rank test was used to assess differences between groups of QTc at follow-up. Cox proportional hazard models were used to explore the risk factors associated with the occurrence of the composite endpoint, and logistic regression models were applied for prolonged QTc. The covariates entered in the model were chosen according to their clinical significance and whether the variable was statistically significant at univariate analysis. All statistical analyses were performed with R version 3.6.1 (R Foundation for Statistical Computing, Vienna, Austria). Statistical significance was set at *p* ≤ 0.05.

## Results

### Baseline characteristics of patients with prolonged baseline QTc

A total of 523 patients were followed up over a mean of 12.90 ± 1.68 months after their initial hospital admission for COVID-19. Baseline characteristics are described in Table 1.

**Table 1:** Comparison of baseline characteristics between hospitalized COVID-19 survivors with and without prolonged QTc on admission.

	All patients (*n* = 523)	QTc baseline <480 ms (*n* = 492)	QTc baseline ≥480 ms (*n* = 31)	*p*-value
**Patients’ clinical characteristics**				
Age, years	62.0 (50.0–75.0)	62.0 (50.0–74.0)	75.0 (62.5–82.0)	**0**.**002**
Sex (women)	219 (41.9%)	209 (42.5%)	10 (32.3%)	0.352
Diabetes	86 (16.4%)	74 (15.0%)	12 (38.71%)	**0**.**001**
Hypertension	225 (43.0%)	202 (41.1%)	23 (74.2%)	**0**.**001**
Dyslipidemia	165 (31.5%)	150 (30.5%)	15 (48.4%)	0.060
Obesity	89 (21.3%)	81 (20.8%)	8 (28.6%)	0.462
Current cigarette smoker	14 (2.7%)	12 (2.4%)	2 (6.5%)	**0**.**026**
CV risk factors	345 (70.7%)	320 (69.9%)	25 (83.3%)	0.173
CAD	31 (5.9%)	24 (4.9%)	7 (22.6%)	**0**.**001**
LVEF (%)	62.0 (58.0–65.0)	62.0 (58.0–65.0)	61.0 (56.0–63.5)	0.584
Atrial fibrillation or flutter	33 (6.3%)	24 (4.9%)	9 (29.0%)	**<0**.**001**
Heart failure	17 (3.3%)	14 (2.9%)	3 (9.7%)	0.073
Mod/severe valve heart disease	13 (2.5%)	10 (2.0%)	3 (9.7%)	**0**.**036**
Stroke	22 (4.2%)	16 (3.3%)	6 (19.4%)	**0**.**001**
COPD	35 (6.7%)	30 (6.1%)	5 (16.1%)	**0**.**048**
CKD	30 (5.7%)	25 (5.1%)	5 (16.1%)	**0**.**026**
Cancer history	60 (11.5%)	54 (11%)	6 (19.4%)	0.151
**Laboratory characteristics**				
Hemoglobin, g/dl	13.8 (12.7–14.7)	13.8 (12.7–14.7)	13.7 (12.4–14.6)	0.462
Lymphocytes, per µl	1.07 (0.77–1.45)	1.07 (0.77–1.42)	1.19 (0.78–1.71)	0.354
Creatinine, mg/dl	0.90 (0.71–1.08)	0.89 (0.71–1.08)	0.96 (0.78–1.45)	**0**.**048**
eGFR, ml/min/1.73m^2^	85.7 (67.6–102)	86.3 (68.9–103)	76.9 (50.4–97.5)	**0**.**033**
Serum lactate, mmol/L	1.29 (1.02–1.60)	1.28 (1.02–1.59)	1.58 (1.14–1.83)	**0**.**027**
CRP, mg/dl	6.50 (2.80–12.6)	6.30 (2.80–12.4)	9.30 (5.95–17.6)	**0**.**038**
Procalcitonin, ng/ml	0.11 (0.07–0.19)	0.11 (0.07–0.18)	0.17 (0.10–0.31)	**0**.**038**
Lactate dehydrogenase, U/L	280 (228–359)	279 (225–358)	310 (245–400)	0.112
D-dimer, ng/ml	640 (420–1035)	630 (420–990)	810 (525–1442)	0.054
NT-proBNP, pg/ml	132 (39.9–348)	120 (37.1–317)	467 (290–2410)	**<0**.**001**

Results are expressed as mean ± SD, median (interquartile range), or number (percentage). Bold *p* values are statistically significant.

COVID-19, coronavirus disease 2019; CAD, coronary artery disease; CKD, chronic kidney disease; COPD, chronic obstruction pulmonary disease; CRP, C-reactive protein; CV, cardiovascular; eGFR, estimated glomerular ﬁltration rate; LVEF, left ventricular ejection fraction; NT-proBNP, N-terminal pro-brain natriuretic peptide.

Patients discharged alive with baseline prolonged QTc were older and had more cardiovascular risk factors, cardiac disease (arrhythmias, coronary artery disease, significant valve heart disease), and comorbidities [previous stroke, chronic obstruction pulmonary disease (COPD), and chronic kidney disease]. Moreover, they had higher C-reactive protein levels, lactate dehydrogenase, procalcitonin, and N-terminal pro-brain natriuretic peptide (NT-proBNP), and lower estimated glomerular ﬁltration rate.

### Long-term clinical impact of prolonged QTc interval in COVID-19 survivors

In COVID-19 survivors, the composite primary endpoint (mortality, hospital readmission, or new arrhythmia) at 1-year follow-up was 10%, primarily due to noncardiac readmission (9.74%). The mortality rate was 0.95%, and only two patients (0.48%) had new atrial fibrillation (AF).

**Figure 2:**
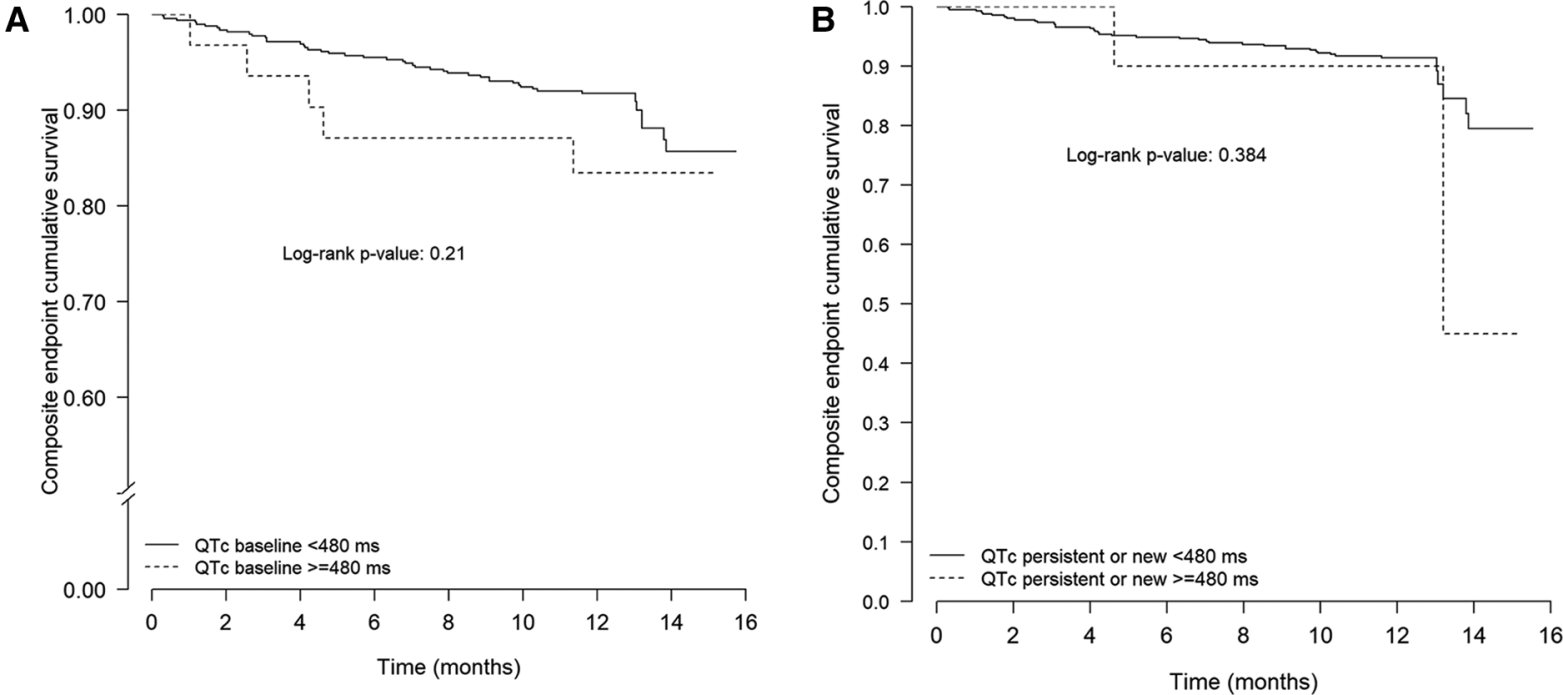
Kaplan–Meier curves for the composite endpoint (death or hospital readmission or new arrhythmia) in 421 COVID-19 survivors according to the presence or absence of persistent or new prolonged QTc (**A**) or persistent or new prolonged QTc at 7 months (**B**). COVID-19, coronavirus disease 2019.

There was no relationship between prolonged QTc (at admission or follow-up) and the composite endpoint at 1 year (Tables 2 and 3 and *<*Figures 2A, B).

**Table 2:** Comparison of clinical, laboratory, and ECG characteristics between COVID-19 survivors with and without the composite endpoint at 1 year after hospital discharge.

	All patients (*n* = 523)	With composite endpoint (*n* = 51)	Without composite endpoint (*n* = 472)	*p*-value
**Patients’ clinical characteristics**				
Age, years	62.0 (50.0–75.0)	75.0 (69.5–82.5)	61.0 (50.0–73.0)	**<0**.**001**
Sex (women)	219 (41.9%)	21 (39.2%)	198 (42.2%)	0.798
Diabetes	86 (16.4%)	18 (35.3%)	68 (14.4%)	**<0**.**001**
Hypertension	225 (43.0%)	33 (64.7%)	192 (40.7%)	**0**.**002**
Dyslipidemia	165 (31.5%)	23 (45.1%)	142 (30.1%)	**0**.**042**
Obesity	89 (21.3%)	8 (21.1%)	81 (21.3%)	1.00
Current cigarette smoker	14 (2.7%)	2 (3.9%)	12 (2.5%)	0.151
CV risk factors	345 (70.7%)	40 (81.6%)	305 (69.5%)	0.108
Ischemic chronic disease	31 (5.9%)	10 (19.6%)	21 (4.5%)	**<0**.**001**
LVEF (%)	62.0 (58.0–65.0)	60.0 (55.0–65.0)	62.0 (58.8–65.0)	0.446
Atrial fibrillation or flutter	33 (6.3%)	8 (15.7%)	25 (5.3%)	**0**.**010**
Heart failure	17 (3.3%)	2 (3.9%)	15 (3.18%)	0.677
Mod/severe valve heart disease	13 (2.5%)	2 (3.9%)	11 (2.3%)	0.367
Stroke	22 (4.2%)	4 (7.8%)	18 (3.8%)	0.257
COPD	35 (6.7%)	6 (11.8%)	29 (6.1%)	0.137
Chronic kidney failure	30 (5.7%)	7 (13.7%)	23 (4.9%)	**0**.**019**
Cancer history	60 (11.5%)	19 (37.3%)	41 (8.7%)	**<0**.**001**
**Laboratory characteristics**				
Hemoglobin, g/dl	13.8 (12.7–14.7)	13.2 (11.8–14.3)	13.8 (12.8–14.7)	**0**.**015**
Lymphocytes, per µl	1.07 (0.77–1.45)	0.88 (0.64–1.56)	1.09 (0.79–1.45)	0.153
Creatinine, mg/dl	0.90 (0.71–1.08)	1.01 (0.68–1.21)	0.89 (0.71–1.07)	0.261
eGFR, ml/min/1.73m^2^	85.7 (67.6–102)	72.2 (57.9–98.9)	86.7 (69.3–103)	**0**.**049**
Serum lactate, mmol/L	1.29 (1.02–1.60)	1.41 (1.10–1.85)	1.28 (1.02–1.59)	0.154
CRP, mg/dl	6.50 (2.80–12.6)	7.20 (4.20–12.8)	6.20 (2.80–12.5)	0.207
Procalcitonin, ng/ml	0.11 (0.07–0.19)	0.10 (0.07–0.24)	0.11 (0.07–0.19)	0.885
Lactate dehydrogenase, U/L	280 (228–359)	279 (224–365)	280 (230–359)	0.880
D-dimer, ng/ml	640 (420–1035)	885 (638–1572)	610 (410–980)	**0**.**001**
NT-proBNP, pg/ml	132 (39.9–348)	317 (185–553)	114 (36.1–325)	**<0**.**001**
**ECG characteristics**				
Baseline QTc ≥ 480 ms	31 (5.9%)	5 (9.8%)	26 (5.5%)	0.212
7-month QTc ≥ 480 ms	10 (2.4%)	2 (4.8%)	8 (2.1%)	0.262

Results are expressed as mean ± SD, median (interquartile range), or number (percentage). Bold *p* values are statistically significant.

ECG, electrocardiogram; COVID-19, coronavirus disease 2019; COPD, chronic obstruction pulmonary disease; CRP, C-reactive protein; CV, cardiovascular; eGFR, estimated glomerular ﬁltration rate; LVEF, left ventricular ejection fraction; NT-proBNP, N-terminal pro-brain natriuretic peptide.

**Table 3:** Adverse events at 1 year after hospital discharge in COVID-19 survivors with and without prolonged QTc at 7 months follow-up.

	All patients (*n* = 421)	7 m QTc < 480 ms (*n* = 415)	7 m new QTc ≥ 480 ms (*n* = 6)	*p*-value
Combined endpoint “Mortality, Hospital readmission and New Arrhythmia”, *N* (%)	42 (10%)	41 (9.88%)	1 (16.7%)	0.470
Mortality, *N* (%)	4 (0.95%)	3 (0.72%)	1 (16.7%)	0.056
Hospital readmission, *N* (%)	41 (9.74%)	40 (9.64%)	1 (16.7%)	0.461
New arrhythmia, *N* (%)	2 (0.48%)	1 (0.24%)	1 (16.7%)	**0**.**028**
	All patients (*n* = 421)	7 m QTc < 480 ms (*n* = 411)	7 m persistent or new QTc ≥ 480 ms (*n* = 10)	*p*-value
Combined endpoint “Mortality, Hospital readmission and New Arrhythmia”, *N* (%)	42 (10%)	40 (9.7%)	2 (20%)	0.262
Mortality, *N* (%)	4 (0.95%)	3 (0.73%)	1 (10%)	0.092
Hospital readmission, *N* (%)	41 (9.74%)	39 (9.49%)	2 (20%)	0.253
New arrhythmia, *N* (%)	2 (0.48%)	1 (0.24%)	1 (10%)	**0**.**047**

Bold *p* values are statistically significant. COVID-19, coronavirus disease 2019.

Independent predictors of the composite endpoint are presented in Table 4. Age, diabetes mellitus, previous coronary artery disease, and cancer were independently related to the composite endpoint at 1 year, whereas prolonged QT was not {hazard ratio (HR) 0.86 [95% confidence interval (CI) 0.231–3.203], *p* = 0.822}.

**Table 4:** Independent predictors of the composite endpoint (death, hospital readmission due to any cause, new arrhythmia) at 1 year after hospital discharge.

	HR	95% CI	*p*-value
Baseline QTc	0.86	(0.231–3.203)	0.822
Age, years	1.07	(1.035–1.107)	**<0**.**001**
Diabetes mellitus	2.937	(1.31–6.584)	**0**.**009**
CAD	2.672	(1.002–7.122)	**0**.**049**
Cancer history	2.68	(1.236–5.808)	**0**.**013**

Bold *p* values are statistically significant. HR, hazard ratio; CI, confidence interval; CAD, coronary artery disease; eGFR, estimated glomerular ﬁltration rate.

### Natural history of QT interval and factors associated with prolonged QT at follow-up

Although the initial protocol established that follow-up would be done at 6 months after the index hospitalization, several COVID waves and in-person visit limitations made that impossible for some patients. Thus, the mean follow-up was 7.9 ± 2.2 months. A subgroup of 421 patients who consented to a repeat ECG were included in this analysis. In this group, 25 (5.9%) patients had a prolonged QTc interval on admission; remarkably, it normalized in most of them (84%) at follow-up. Only 2.4% (10 patients) had a prolonged QTc at follow-up; 6 cases had a new prolonged QTc (from normal after initial admission to prolonged QTc at follow-up) and 4 had persistent QTs (already prolonged at baseline and continued to be prolonged at follow-up) (Table 5).

**Table 5:** QTc interval analysis at hospital admission and 7 months after COVID-19 survivors hospital discharge.

QT characteristics	Baseline QTc (*n* = 523)	7-month QTc (*n* = 420)	*p*-value
Mean QTc (ms)	432.63 ± 27.67	409.24 ± 29.20	**<0.001**
QTc ≥480 ms	31 (6.3%)	10 (2.4%)	**0.007**
QT evolution	7-month QTc < 480 ms	7-month QTc ≥ 480 ms	Total
Baseline QTc <480 ms	390 (92.6%)	6 (1.43%)	396
Baseline QT ≥480 ms	21 (5%)	4 (1%)	25
Total	411 (97.6%)	10 (2.4%)	421

Bold *p* values are statistically significant. COVID-19, coronavirus disease 2019.

Prolonged QTc was deﬁned as a QTc ≥ 480 ms (18). A baseline QTc *<*480 ms and QTc ≥480 at 8 months was named “new prolonged QTc at follow-up.” A baseline QTc ≥ 480 ms, which continued to be prolonged (QTc ≥ 480 ms) at 8 months, was named “persistent prolonged QTc at follow-up.” A baseline QTc ≥ 480 ms, which reverted to normal (QTc ≤ 480 ms) at 8 months, was named “normalized QTc at follow-up.”

Supplementary Table S1 describes the baseline characteristics, blood tests, and treatment of patients with QTc prolongation at follow-up.

The independent predictors of QTc prolongation at follow-up were the presence of obesity, coronary artery disease, chronic obstructive pulmonary disease, and cancer (Table 6).

**Table 6:** Independent predictors of prolonged QT at follow-up.

Prolonged QTc at follow-up	OR	95% CI	*p*-value
Obesity	7.955	(1.836–40.057)	**0**.**007**
CAD	7.295	(1.268–36.166)	**0**.**016**
COPD	8.004	(1.395–40.02)	**0**.**012**
Cancer history	5.369	(1.21–22.369)	**0**.**02**

Bold *p* values are statistically significant. OR, odds ratio; CI, confidence interval; CAD, coronary artery disease; COPD, chronic obstruction pulmonary disease.

At 1-year follow-up, only new atrial fibrillation occurred more often in patients with new prolonged QTc at follow-up (16.7% vs. 0.24%, *p* = 0.028) (Table 3).

## Discussion

Our study is a prospective registry of 523 patients with the aim of evaluating the natural history of QTc interval and its clinical impact on 1-year prognosis in COVID-19 survivors. Results showed that a prolonged QTc on admission had no clinical impact on the long-term follow-up of patients who survived hospitalization due to COVID-19. It normalized in most patients at 7 months after hospital discharge. Few patients demonstrated a new prolonged QTc at follow-up, and it was not associated with inflammatory markers or adverse events. Patients with prolonged QTc at follow-up had higher rates of atrial fibrillation at 1-year follow-up. The presence of obesity, COPD, coronary artery disease, and cancer were independently related to prolonged QTc at follow-up.

QTc interval prolongation occurs when a significant proportion of cells in the ventricular myocardium experience a reduction of outward repolarizing currents or an increase of inward currents, creating a prolongation of the action potential (18). This condition is characterized by both QTc prolongation and T wave abnormalities. The congenital form of long QTc syndrome is uncommon. However, acquired QTc prolongation is much more common and linked to different issues, especially in sick and hospitalized patients (19, 20).

### Clinical impact of QT prolongation on COVID-19 survivors

The effect of acute SARS-CoV-2 or other acute infections on QTc prolongation and its relationship with a worse short-term prognosis is well established (5, 20, 21). However, little is known about the mid- and long-term QTc evolution after acute infection and its implications at follow-up. In a case series of four patients, Beer et al. reported transient marked QTc prolongation and ventricular arrhythmias in the setting of acute infection and inflammation. Prolonged QTc normalized after patients overcame the infection, but the medium- to long-term prognosis and complications were not explored (22). In our study, most of the COVID-19 survivors with a prolonged QTc interval on admission demonstrated normalization on the control ECG at follow-up. We did not observe a relationship between prolonged QTc at admission and the composite endpoint at 1 year. Assuming that QTc prolongation is a reflection of severity, the mechanisms that caused the QTc prolongation in patients who recovered from an acute infection would be at least partially resolved. The logical conclusion under this assumption would be that the basal QTc would not have a long-term influence on the prognosis of our patients, as we have reported in our study.

### Natural history of QT interval and factors related to prolonged QT at follow-up

A significant relationship between systemic inflammation and QTc prolongation during the acute phase of infection has been demonstrated in COVID-19 and non-COVID-19 patients (6, 7, 22, 23). A recent study in patients with active severe COVID-19 showed that QTc was significantly prolonged if IL-6 levels were high, and it rapidly normalized in correlation with decrease in IL-6 (24).

In addition, some conditions have been associated with acquired prolonged QTc in outpatients. Previous reports have shown systemic inflammation and autoimmune diseases as factors that are crucially involved in ventricular repolarization (18). An independent association between C-reactive protein levels and QTc prolongation has been shown, even in apparently healthy subjects or in noninflammatory heart diseases like hypertension, coronary artery disease, or Takotsubo cardiomyopathy (25–27).

Given the evidence, if QTc alterations might be related to a pro-inflammatory state, it seems logical to think that overcoming acute coronavirus disease will lead to QTc normalization. Accordingly, in our study, most of the COVID-19 survivors with a prolonged QTc interval on admission demonstrated normalization in the control ECG at follow-up. It seems that abnormal acute ventricular repolarization observed in the setting of COVID-19 infection self-resolved as the viral illness course remitted. Banai et al*.* carried out a study in which prolonged QTc in hospitalized COVID-19 patients was associated with disease severity, myocardial injury, and 1-year mortality (28). However, they did not exclude patients who had died from COVID-19 in their analysis. In our study, we analyzed only patients who had survived SARS-CoV-2 infection and hospital admission, suggesting that our patients had completely overcome COVID-19.

In our study, the independent predictors of prolonged QTc at follow-up in COVID-19 survivors were the presence of obesity, previous coronary artery disease, COPD, and cancer. Patients affected by these diseases likely have some degree of endothelial dysfunction and chronic inflammation related to an increased level of circulating cytokines. The putative cytokine-mediated mechanisms are complex and include direct actions on cardiomyocyte ion channel function and indirect effects resulting from sympathetic activation (29).

### QTc and AF

As explained above, the presence of chronic diseases, such as obesity, COPD, coronary artery disease, and cancer, was related to prolonged QTc at follow-up. However, it is well known that these factors are also predictors of AF in the general population (30, 31). Interestingly, we observed a higher rate of new atrial fibrillation in patients with new prolonged QTc at follow-up (16.7% vs. 0.24%, *p* = 0.028). However, it was not significant in the multivariate regression model. Several studies have shown that individuals with a prolonged QTc interval have a high risk of developing atrial fibrillation (32, 33), although the pathophysiology of the ventricular action potential and its relationship with AF are not fully understood. Furthermore, a recent study has described a higher incidence of atrial fibrillation at 12-month follow-up in COVID-19 survivors (15).

It is possible that the QT interval was a marker of other AF risk factors, which might explain why prolonged QT was not found as an independent predictor in the multivariate regression model.

### Potential medication with a known effect on the QT interval

It is important to note that potential medication with a known effect on the QT interval was registered in the acute phase and at follow-up. In the acute phase, we only included patients in whom ECG was taken within the ﬁrst 48 h after laboratory-conﬁrmed COVID-19 diagnosis and before the administration of any medication with a known effect on the QT interval. We did not find any significant relation between patient’s treatment and prolonged QTc at follow-up (Supplementary Table S1).

### The role of the electrocardiogram in post-COVID patients

Persistent COVID, i.e., persistent symptoms and late organ damage in patients who have suffered from COVID-19, is an entity that affects a substantial percentage of post-COVID-19 patients. Studies are being carried out to learn more about this syndrome, since it diminishes patients’ quality of life. Pulmonary sequelae have been established (34), but studies have also shown that cardiac sequelae could be a relevant topic. In a systematic review about cardiac sequelae after COVID-19, Ramadan et al*.* noted that survivors had a higher chance of developing heart failure, arrhythmias, and myocardial infarction (35). Considering the above arguments, ECG could be a widely available, safe, and cheap tool that could help detect patients at risk of developing arrhythmias or other cardiovascular diseases. However, we have not been able to prove the ability of QTc to predict long-term adverse events in COVID-19 survivors.

## Limitations

Our study has some limitations. The study was performed in a single center and thus may not have been representative of the overall population. The small number of patients with prolonged QTc at follow-up limited the statistical power and number of conclusions that could be extracted. A more extended follow-up to collect more data would be very helpful. Moreover, we did not collect data on electrolyte levels in our patients, and this could be a limitation in interpreting the results of our study. We were not able to monitor our patients with 24 h Holter monitoring or other kinds of cardiac-rhythm monitoring devices during follow-up. We only collected clinical episodes of arrhythmia, and we did not register subclinical arrhythmic events, which could have been a limitation of our study.

## Conclusions

Acute prolonged QTc observed in the setting of COVID-19 infection self-resolved in most COVID-19 survivors at 6 months after hospital discharge. Prolonged QTc on admission had no clinical impact on the long-term follow-up. QTc prolongation in COVID-19 survivors was not related to adverse events at 1 year. Notably, few patients showed QTc prolongation at follow-up; QTc prolongation was independently related to obesity and chronic disease.

## Data Availability

The raw data supporting the conclusions of this article will be made available by the authors, without undue reservation.
