# Supplementary material for: The natural history of QTc interval and its clinical impact in coronavirus disease 2019 survivors after 1 year
Source: Front Cardiovasc Med. 2023 Apr 6;10:1140276. doi: 10.3389/fcvm.2023.1140276 (PMC10117953; doi:10.3389/fcvm.2023.1140276)
Supplement: Supplementary file 1 [file Table1.docx]

**Supplemental Table 1: Comparison of demographic, treatment and laboratory characteristics between hospitalized COVID-19 survivors with and without prolonged QTc at 7 months follow-up.**

|  | **All patients**  **(n = 421)** | **QTc 7m <480 ms**  **(n = 411)** | **QTc 7m ≥ 480 ms**  **(n = 10)** | **p-Value** |
| --- | --- | --- | --- | --- |
| **Patients’ clinical characteristics** |  |  |  |  |
| Age, years | 63.0 [51.0;74.0] | 62.0 [51.0;74.0] | 74.5 [67.2;75.8] | **0.034** |
| Sex (women) | 176 (41.8%) | 174 (42.3%) | 2 (20.0%) | 0.204 |
| Diabetes | 67 (15.9%) | 66 (16.1%) | 1 (10.0%) | 1.000 |
| Hypertension | 174 (41.3%) | 167 (40.6%) | 7 (70.0%) | 0.100 |
| Dyslipidemia | 137 (32.5%) | 133 (32.4%) | 4 (40.0%) | 0.734 |
| Obesity | 68 (20.1%) | 63 (19.1%) | 5 (50.0%) | **0.031** |
| Current cigarette smoker | 13 (3.09%) | 13 (3.16%) | 0 (0.00%) | 0.446 |
| CV Risk factors | 276 (70.2%) | 266 (69.5%) | 10 (100%) | **0.037** |
| CAD | 26 (6.18%) | 23 (5.60%) | 3 (30.0%) | **0.019** |
| LVEF (%) | 61.0 [56.8;65.0] | 61.0 [57.0;65.0] | 59.0 [54.5;62.0] | 0.547 |
| Atrial fibrillation or flutter | 29 (6.89%) | 26 (6.33%) | 3 (30.0%) | **0.025** |
| Heart failure | 13 (3.09%) | 13 (3.16%) | 0 (0.00%) | 1.000 |
| Mod/severe valve heart disease | 12 (2.85%) | 12 (2.92%) | 0 (0.00%) | 1.000 |
| Stroke | 16 (3.80%) | 16 (3.89%) | 0 (0.00%) | 1.000 |
| COPD | 29 (6.89%) | 26 (6.33%) | 3 (30.0%) | **0.025** |
| CKD | 24 (5.70%) | 22 (5.35%) | 2 (20.0%) | 0.116 |
| Cancer history | 50 (11.9%) | 46 (11.2%) | 4 (40.0%) | **0.022** |
| **Treatment at 7m follow-up** | 145 (34.4%) | 139 (33.8%) | 6 (60.0%) | 0.100 |
| Beta-Blockers | 64 (15.2%) | 60 (14.6%) | 4 (40.0%) | 0.050 |
| Amiodarone | 5 (1.19%) | 4 (0.97%) | 1 (10.0%) | 0.114 |
| IC antiarrhythmic | 5 (1.19%) | 5 (1.22%) | 0 (0.00%) | 1.000 |
| Psychiatric drugs | 98 (23.3%) | 95 (23.1%) | 3 (30.0%) | 0.704 |
| **Laboratory characteristics** |  |  |  |  |
| Hemoglobin, g/dl | 13.4 ± 1.39 | 13.4 ± 1.38 | 13.6 ± 1.87 | 0.834 |
| Lymphocytes, per $\mu$L | 2020 [1700;2515] | 2040 [1710;2490] | 1980 [1605;2535] | 0.838 |
| Creatinine, mg/dL | 0.96 [0.80;1.14] | 0.95 [0.79;1.14] | 1.10 [0.93;1.28] | 0.195 |
| eGFR, mL/min/1.73m2 | 76.0 (22.3) | 76.3 (22.2) | 68.6 (25.4) | 0.495 |
| CRP, mg/dL | 0.16 [0.10;0.35] | 0.16 [0.10;0.36] | 0.14 [0.12;0.25] | 0.805 |
| D-dimer, ng/mL | 310 [190;448] | 320 [190;450] | 220 [190;300] | 0.461 |
| NT-proBNP, pg/mL | 107 [46.6;171] | 104 [45.9;163] | 199 [112;838] | 0.084 |

Results are expressed as mean ± standard deviation, median and [interquartile range] or number and (percentage). BB: betablockers. CAD: coronary artery disease. CKD: chronic kidney disease. COPD: chronic obstruction pulmonary disease. CRP: C-reactive protein. CV: cardiovascular. eGFR: estimated glomerular ﬁltration rate. LVEF: left ventricular ejection fraction.
